# Barriers to brain health behaviours: results from the Five Lives Brain Health Ireland Survey

**DOI:** 10.3389/fpsyg.2023.1101514

**Published:** 2023-08-24

**Authors:** Tim Dukelow, Philip Vassilev, Erin Grace Lawrence, Liron Jacobson, Ivan Koychev, Kinan Muhammed, Sean P. Kennelly

**Affiliations:** ^1^Cork University Hospital (CUH), Cork, Ireland; ^2^Five Lives SAS, Tours, France; ^3^Unit of Psychological Medicine, Centre for Psychiatry and Mental Health, Wolfson Institute of Population Health, Queen Mary, University of London, London, United Kingdom; ^4^Department of Psychiatry, University of Oxford, Oxford, England, United Kingdom; ^5^Nuffield Department of Clinical Neurosciences, University of Oxford, Level 6, West Wing, John Radcliffe Hospital, Oxford, England, United Kingdom; ^6^Department of Age Related Health Care, Tallaght University Hospital, Dublin, Ireland; ^7^Trinity College Dublin, Dublin, Ireland

**Keywords:** brain health, risk reduction, behavioural change, dementia prevention, lifestyle modification

## Abstract

Modifiable risk factors for dementia remain prevalent in Ireland. A detailed examination of barriers to risk reduction behaviours in an Irish context has heretofore been lacking. Many existing studies examining barriers to brain health behaviours fail to examine how they might vary across different modifiable risk factors. This study undertook a detailed assessment of barriers to individual risk reduction behaviours. As existing research suggests that barriers may vary across sociodemographic factors, we sought to investigate the distribution of barriers across age, gender, educational status, and household income. The Five Lives Brain Health Ireland Survey is a cross-sectional survey that was distributed online amongst a non-patient population. The survey captured the following: (1) Sociodemographic factors; (2) Barriers to brain health behaviours; (3) Exposure to, and knowledge of, modifiable risk factors for dementia, namely diet, social interaction, exercise, hypertension, sleep, current low mood/depression, current smoking, alcohol consumption, cognitive stimulation, hearing impairment, diabetes, air pollution, and head injury; (4) Participants’ perceptions regarding potential for dementia prevention, and risk reduction. Lack of motivation was the most prevalent barrier to consuming a healthy diet (64%, *n* = 213), physical activity (77.7%, *n* = 167), smoking cessation (68%, *n* = 85), and moderation of alcohol intake (56.3%, *n* = 67). Practical factors were the most prevalent barriers to addressing low mood (56.5%, *n* = 87), air pollution (30.1%, *n* = 58), hearing impairment (63.8%, *n* = 44), diabetes (11.1%, *n* = 5), and head injury (80%, *n* = 8). Emotional factors were the most prevalent barriers to engaging in mentally stimulating activity (56.9%, *n* = 66), social activity (54.9%, *n* = 302), and good sleep (70.1%, *n* = 129). Lack of knowledge was the most prevalent barrier to hypertension control (14.4%, *n* = 29). Distribution of barriers varied across age, gender, educational status, and household income. This study investigated barriers to lifestyle change to improve brain health in an Irish sample of adults aged 50 and above. Detailed subtyping of barriers, as well as examination of differences according to age, gender, education, and income were undertaken. The heterogeneity of barriers to brain health behaviours revealed in this study highlights the necessity to tailor public health interventions to their target population, taking into account the gender, age, educational status, and income of recipients.

## 1. Introduction

The number of people living with dementia globally is increasing. Estimated dementia prevalence is projected to increase from 57·4 million cases globally in 2019 to 152·8 million cases in 2050 (GBD 2019 Dementia Forecasting Collaborators, 2022). Negative stereotypes of the condition predominate (Low and Purwaningrum, 2020), and dementia has been cited as one of the most feared conditions amongst members of the public (MetLife Foundation, 2011; Kessler et al., 2012). Dementia research funding is increasing, with spending doubling in many Western countries between 2011 and 2016 (Pickett and Brayne, 2019). The importance of research into dementia prevention, and risk reduction is emphasised in the WHO’s Global action plan on the public health response to dementia 2017–2025 (WHO, 2017).

Burgeoning evidence supports the potential for dementia prevention globally. The Lancet Dementia Commission identified 12 modifiable risk factors which account for 40% of dementias globally (Livingston et al., 2020). The population attributable fraction for modifiable dementia risk factors is higher in low-and middle-income countries where certain risk factors are more prevalent (Livingston et al., 2020). Less childhood education, smoking, hypertension, obesity, and diabetes are, for example, more prevalent in China, India, and certain Latin American countries as compared to high income countries (Mukadam et al., 2019). Whilst improvements in cardiovascular risk reduction in particular may account for falling dementia incidence in Western Society (Larson et al., 2013; Wolters et al., 2020), these trends have not been replicated in Low-and Middle-Income Countries (LMICs; Li et al., 2007; Wu et al., 2018).

It is recognised that cognition exists on a spectrum encompassing normal cognition, subjective cognitive decline, mild cognitive impairment (MCI), and dementia (Knopman et al., 2015). People with MCI have cognitive impairment beyond that expected for age and education but remain functionally unimpaired (Petersen et al., 1999, 2021). The entities of subjective cognitive decline and MCI have represented opportune stages at which to intervene from a risk reduction perspective. Specific risk reduction strategies for those with MCI, as well as normal cognition, are set out in the World Health Organisation Risk Reduction of Cognitive decline and dementia guidelines (World Health Organization, 2019). In clinical settings, such risk reduction is increasingly addressed in a personalised and protocolised fashion. Evolving brain health services are likely in time to fulfil a number of roles, including risk profiling, risk communication, and risk reduction (Altomare et al., 2021). Furthermore, large scale, multidomain interventions have demonstrated the potential to benefit cognition in those at risk of dementia (Kivipelto et al., 2018). Particular efforts are ongoing to harmonise lifestyle intervention studies based on the FINGER study globally (Kivipelto et al., 2020). Such ‘high risk’ prevention strategies may however have limited impact at population level (Rose, 2001). Whilst secondary prevention in those with, or indeed seeking, a diagnosis is important, shifting the focus to encompass primary prevention in the general population is vital if dividends are to be seen at a population level (Milne et al., 2021). Population based strategies are radical and may confer a large benefit (Rose, 2001). Increasing awareness of modifiable risk factors for dementia represents a core function of dementia awareness campaigns (WHO, 2017). Indeed, lack of knowledge has been cited as the main barrier to behavioural change in dementia risk reduction (Curran et al., 2021). Barriers to engaging in risk reduction behaviours are numerous and diverse. In addition to lack of knowledge, existing research suggests that barriers might include lack of motivation, lack of time, organisational issues, financial reasons, health problems, and other factors (Heger et al., 2019; Curran et al., 2021; Van Asbroeck et al., 2021). In this context, whether risk reduction strategies are implemented on an individual or population level, it is uncertain if increasing awareness of dementia risk factors alone will be sufficient to engender behavioural change. Addressing barriers to dementia risk reduction is thus essential.

As illustrated elsewhere in this issue, modifiable risk factors for dementia remain prevalent in Ireland (Dukelow et al., 2023). A detailed examination of barriers to risk reduction behaviours in an Irish context has heretofore been lacking. More broadly, many existing studies examining barriers to brain health behaviours consider barriers generically, failing to examine how they might vary across different modifiable risk factors for dementia. In this context, this study aimed to undertake a detailed assessment of barriers to individual risk reduction behaviours. As existing research suggests that barriers to brain health behaviours may vary across sociodemographic factors (Heger et al., 2019; Van Asbroeck et al., 2021), we sought to investigate the distribution of barriers across factors such as age, gender, educational status, and household income.

## 2. Methods

### 2.1 Study design and participants

The Five Lives Brain Health Ireland Survey (FLBHIS) is a cross-sectional survey that was distributed online amongst an Irish non-patient population. The FLBHIS was developed through an iterative process. Informed by current literature and expert opinion, a 100-question survey was devised (see Appendix 1). The survey was adapted from the Lifestyle Barriers for Cognitive Health Questionnaire (K. Muhammed 2021, personal communication, 2 September) to be suitable for an Irish population. Risk factors were derived from the 2020 report of the Lancet Commission on Dementia prevention, intervention, and care (Livingston et al., 2020). Additional items relating to other modifiable risk factors for cognitive decline such as sleep issues (Spira et al., 2014) and mental stimulation (Krell-Roesch et al., 2019) were also added to the survey. The barriers used in our study were derived and adapted from the behaviour change wheel, specifically from the COM-B model (Michie et al., 2011). At the heart of this model are three essential conditions, namely capability, opportunity, and motivation. From this model, five subcategory barriers were derived specifically to cover the risk factors for brain health. Conditions were developed and subcategories defined as follows: capability (emotional factors, practical factors, and lack of knowledge), opportunity (social factors), and motivation (lack of motivation). Subcategories related to capability differ from those set out by Michie who distinguished between physical and psychological capability (Michie et al., 2011). Questions were constructed in order to elicit information related to each subcategory. Examples of questions/statements related to smoking and its associated barriers, for example, include the following: ‘*I want to stop smoking but I have not got the willpower to stop on my own*’ (motivation-lack of motivation); ‘*I do not think I could stop smoking due to the social life I have*’ (opportunity-social); and ‘*Stress and pressures from work or personal life mean I cannot quit smoking’* (capability-emotional). This approach allowed the main sources of behaviour to be captured at a higher level whilst allowing more granular subcategorisation where possible.

Inclusion criteria comprised people aged over 50. Participants were excluded if they had a diagnosis of dementia, or had worked in the healthcare sector. A pilot version of the survey was undertaken in January 2022 (*n* = 50). The survey pilot revealed a tendency for respondents to ‘straight-line’ answers, potentially indicating respondent satisficing (Reuning and Plutzer, 2020) or indeed respondent fatigue related to repetitive questions in the original survey design. Phraseology and number of questions were modified accordingly. The modified survey was subsequently administered to a further 555 volunteers in February 2022. A professional market research company, CDR Insights & Analytics Limited, was responsible for administration of the survey online to an existing market research panel. Choice of sample size was guided by logistical constraints and review of comparable literature (Heger et al., 2019). With regard to the sampling technique, a 1:1 male to female gender split was pre-specified in order to approximately reflect the Irish population. No upper age-limit was pre-determined.

Prior to commencement, this study was reviewed by The St. James’ Hospital/Tallaght University Hospital Joint Research Ethics Committee who advised that, in keeping with local institutional and legislative requirements, formal approval was not necessary.

### 2.2. Measures

The survey captured the following information: (1) Sociodemographic factors (gender, age, ethnicity, household income and level of education); (2) Barriers to brain health behaviours; (3) Exposure to, as well as knowledge of modifiable risk factors for dementia, namely diet, social interaction, exercise, hypertension, sleep, current low mood/depression, current smoking, alcohol consumption, cognitive stimulation, hearing impairment, diabetes, air pollution and head injury; (4) Participants’ perceptions regarding potential for dementia prevention and risk reduction.

Alcohol intake (>14 units per week), being overweight, smoking, low physical activity, sleep quality, depression/low mood, low social interaction, low mental stimulation and residing in an area with high air pollution were self-reported risk factors, whereas diabetes, hypertension and hearing impairment were defined as being diagnosed by a healthcare professional.

### 2.3. Statistical analysis

All personal data were removed from the dataset prior to receipt by the research team. Analyses were conducted using IBM SPSS Statistics software. Frequency counts and percentages were used to show rates of exposure to modifiable risk factors for dementia amongst the sample. For reporting and analysis purposes, barriers were broadly classified as relating to lack of knowledge, lack of motivation, emotional factors, social factors and practical factors. Frequency counts and percentages were used to illustrate prevalence of barriers relevant to individual risk factors. For all analyses, statistical significance was defined as *p* < 0.05.

Z-tests with a Bonferroni correction for multiple comparisons were utilised to examine distribution of barriers between groups. Significant differences were confirmed using chi-square tests. Age was categorised into two groups, defined as 50–64 years old, and 65 years and above for the purpose of analysis. Household income was categorised into two groups, defined as ≤€40,000 per year, and > €40,000 per year. Due to small numbers in this group (*n* = 3), those who responded that they would prefer not to disclose their gender were excluded from the dataset for analyses. Due to small numbers who had been educated to primary school level (*n* = 10), this group was combined with those who had been educated to secondary school level for the purpose of analysis. People who chose not to disclose their household income (*n* = 55) were excluded from analyses including the household income variable. Due to low numbers which precluded Z-tests, blood pressure, hearing impairment, diabetes and head injury were excluded from the analysis of sociodemographic factors as predictors of barrier prevalence.

A two-step cluster analysis was used to classify participants in distinct groups based on age, gender, education and household income. The distance measure was log-likelihood and the clustering criterion was Schwartz’s Bayesian Criterion. Initially, the automatic clustering algorithm of SPSS produced three clusters based on these settings but the cluster quality was classified as ‘Fair’ (average Silhouette measure of cohesion and separation = 0.4). To improve the cluster quality, the number of clusters was increased manually by a factor of 1 until reaching the lowest number of clusters for which a ‘Good’ cluster quality was achieved.

The percentages of people reporting barriers to alcohol use represent a fraction of those exposed to greater than 14 units of alcohol per week. For the barriers to preventing all other risk factors, the percentages refer to the fraction of people who reported exposure to the respective risk factor.

## 3. Results

### 3.1. Sample characteristics

The study population ultimately comprised 551 participants. Of the 555 volunteers to whom the survey was originally administered, three participants were excluded as they did not disclose their gender. One further participant was excluded due to an incomplete dataset. Table 1 outlines the sociodemographic characteristics of the sample. The study population comprised 551 participants with an almost equal proportion of male and female respondents (50.3% male; 49.6% female). Mean age was 59.7 years, with the majority of the sample ranging between 50 and 59 years of age (54.3%). 98.9% of respondents were of White ethnicity. Most participants were educated to secondary school level or higher (98.2%), were employed or self-employed (52.2%), and cohabited with one or more persons (74.4%).

**Table 1:** Sociodemographic characteristics of the sample.

Characteristic	*n* (%)
Age in years	
50–64	437 (79.3)
65 and above	114 (20.7)
Gender	
Male	277 (50.3)
Female	274 (49.7)
Educational attainment	
Primary school	10 (1.8)
Secondary school	282 (51.3)
Undergraduate degree	196 (35.6)
Postgraduate degree	62 (11.3)
Household income level	
Less than €20 000	104 (18.9)
€20 000–€40 000	189 (34.4)
€40 000–€60 000	91 (16.6)
€60 000–€80 000	63 (11.5)
Above €80 000	48 (8.7)
Employment status	
Employed or self-employed	287 (52.2)
Unemployed	91 (16.6)
Retired	172 (31.3)
Home circumstances	
Living alone	141 (25.6)
Living with one other person	197 (35.8)
Living with more than one person	212 (38.6)

Total sample (*N* = 551). Not all totals for each variable sum to 551 due to missing data.

### 3.2. Risk factor exposure and barrier prevalence for individual risk factors

Table 2 outlines exposure to modifiable risk factors, as well as the prevalence of different barriers across individual risk factors. Various practical barriers, and lack of motivation were the most prevalently cited barriers across multiple risk factors. Lack of motivation was the most prevalent barrier to consuming a healthy diet (64%, *n* = 213), physical activity (77.7%, *n* = 167), smoking cessation (68%, *n* = 85), and moderation of alcohol intake (56.3%, *n* = 67). Practical factors were the most prevalent barriers to addressing low mood (56.5%, *n* = 87), air pollution (30.1%, *n* = 58), hearing impairment (63.8%, *n* = 44), diabetes (11.1%, *n* = 5), and head injury (80%, *n* = 8). Emotional factors were the most prevalent barriers to engaging in mentally stimulating activity (56.9%, *n* = 66), social activity (54.9%, *n* = 302), and good sleep (70.1%, *n* = 129). Lack of knowledge was the most prevalent barrier to hypertension control (14.4%, *n* = 29).

**Table 2:** Frequency of barriers across risk factors.

Risk factor	Exposed (% total)	Exposed (n total)		Lack of motivation	Emotional	Social	Practical	Lack of knowledge			Barriers (% from exposed to risk factor)							
**Unhealthy diet**	60.4%	333	I have no motivation	Healthy foods do not satisfy my appetite	Stress from work / personal life	Healthy foods take too long to prepare	Healthy foods cost too much	Not like the taste of healthy foods	I am unsure about what a healthy diet is	people I live with
		%	64.0%	42.6%	37.5%	31.8%	31.2%	26.7%	26.4%	21.9%
		n	213	142	125	106	104	89	88	73
**Low social interaction**	54.8%	302	Do not socialise much as I like being by myself	Worried about COVID-19	Not many friends	No access to facilities to meet	Live too far away	Cannot afford the costs	Stress meeting others	Not enough time
		%	54.9%	49.7%	49.0%	47.0%	46.4%	39.4%	35.1%	24.5%
		n	302	150	148	142	140	119	106	74
**Low physical activity**	39.0%	215	No drive to do it	Gym too expensive	No access to facilities	Physical disability	Home commitments	Work commitments		
		%	77.7%	45.1%	34.4%	33.0%	30.2%	22.3%		
		n	167	97	74	71	65	48		
**Hypertension**	36.7%	202	Would not know how to change my lifestyle	Would not be able to get health support	Would be worried of the outcome of checking BP					
		%	14.4%	9.9%	3.0%					
		n	29	20	6					
**Poor quality sleep**	33.4%	184	Too stressed	Do not know why	Family commitments	Caffeine intake	Work commitments	Noisy area		
		%	70.1%	64.7%	18.5%	17.4%	12.0%	10.9%		
		n	129	119	34	32	22	20		
**Low mood and depression**	27.9%	154	Life at home	Financial worries	Health worries	Worried about asking others	Bad previous experiences with health professionals	Work related stress	Not know how to access services	
		%	56.5%	49.4%	48.7%	37.0%	33.8%	25.9%	23.4%	
		n	87	76	75	57	52	40	36	
**Smoking**	22.7%	125	Not have the willpower to stop	Stress from work or personal life	Other people I live with continue to smoke	Social life I have				
		%	68.0%	46.4%	42.4%	24.8%				
		n	85	58	53	31				
**Alcohol consumption**	21.6%	119	Not feeling I need to reduce alcohol consumption	Alcohol helps me cope with stress	People around me continue to drink	Social life I have				
		%	56.3%	35.3%	20.2%	16.8%				
		n	67	42	24	20				
**Low mental stimulation**	21.1%	116	Not entertaining	No close friends	Lack of time	Financial means				
		%	56.9%	42.2%	36.2%	34.5%				
		n	66	49	42	40				
**Air pollution**	15.1%	83	Could afford to change the area I live or work in							
		%	30.1%							
		n	58							
**Hearing impairment**	12.5%	69	Too high cost	Worried perception of hearing aid	Worried about assessment outcome	Too busy for assessment	Not know how to do assessment			
		%	63.8%	20.3%	10.1%	8.7%	2.9%			
		n	44	14	7	6	2			
**Diabetes**	8.2%	45	Forget to take medication	No time to monitor sugar levels	Sugar levels not well controlled	Worried if problem found	Not know how to seek advice			
		%	11.1%	11.1%	11.1%	6.7%	4.4%			
		n	5	5	5	3	2			
**Activities with risk of head injury**	1.8%	10	Activities that increase the risk of head injury are part of my life	I enjoy taking part in contact sports too much to stop doing them						
		%	80.0%	70.0%						
		n	8	7						

Figures in red indicate small sample sizes excluded from further analyses.

### 3.3. Overall barrier prevalence

**Figure 1:**
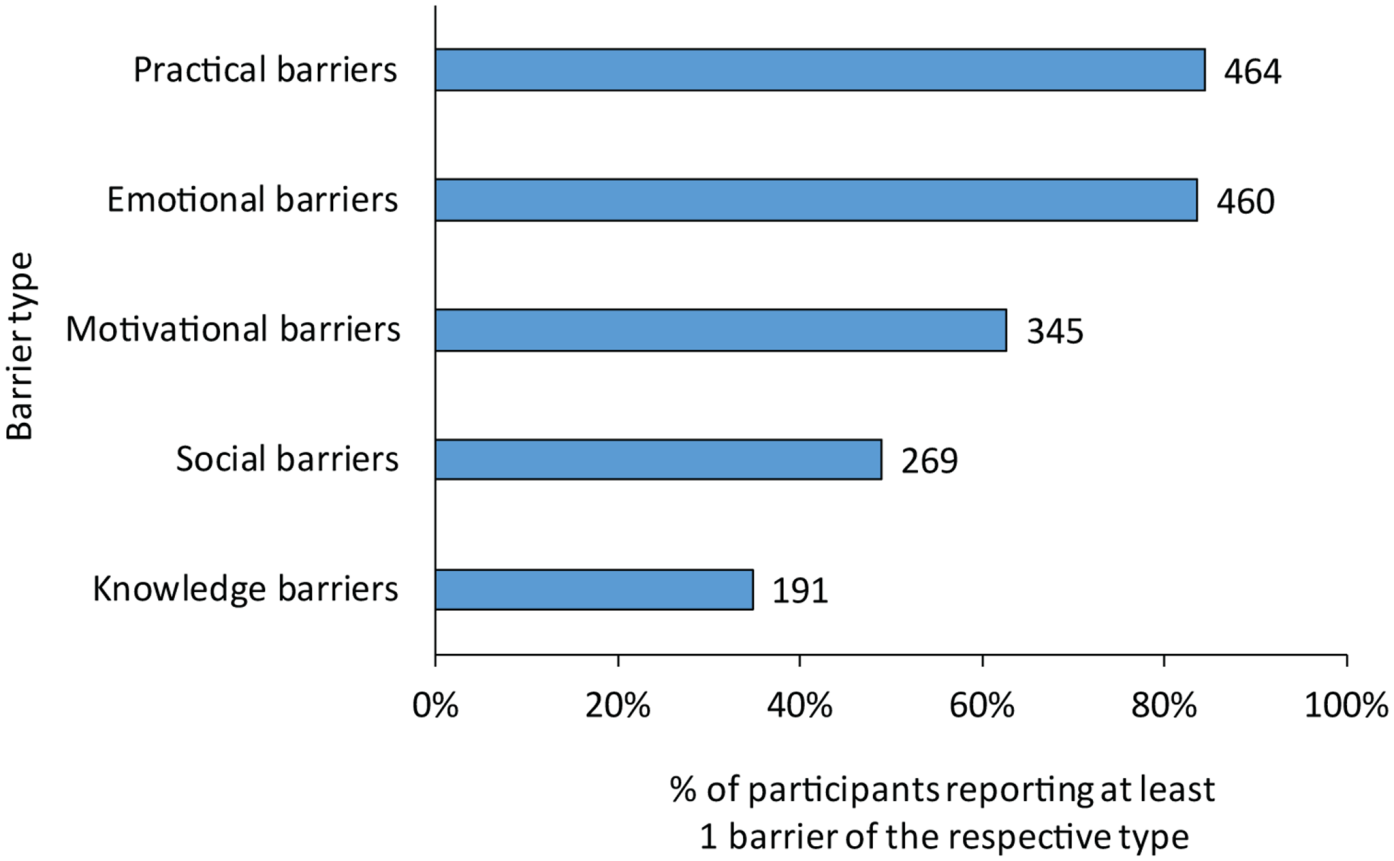
Number and proportion of participants reporting at least one barrier of each subtype.

Figure 1 illustrates the proportion of participants reporting at least one barrier of each subtype. Practical barriers were most commonly reported, with at least one practical barrier reported by 84.2% of the study population (*n* = 464). One or more emotional barriers were reported by 83.5% of the population (*n* = 460), motivational barriers by 62.6% (*n* = 345), and social barriers by 48.8% (*n* = 269). Knowledge barriers were least commonly reported with 34.7% of participants reporting one or more knowledge barriers (*n* = 191).

### 3.4. Effect of gender on exposure to barriers

Table 3 illustrates the effect of gender on exposure to barriers. Females were significantly more likely to report stress from their work and personal life [𝝌^2^(1) = 5.72, *p* = 0.017], and people they live with [𝝌^2^(1) = 9.03, *p* = 0.003] as barriers to consuming a healthy diet. Males were more likely to report not having many friends [χ^2^(1) = 6.46, *p* = 0.011], and living too far away [χ^2^(1) = 5.18, *p* = 0.023] as barriers to engaing in social activity. Females were more likely to report the gym being too expensive as a barrier to engaging in physical activity [χ^2^(1) = 3.87, *p* = 0.049] Male participants were more likely to report caffeine intake [𝝌^2^(1) = 4.77, *p* = 0.029], work commitments [𝝌^2^(1) = 9.97, *p* = 0.002] and residing in a noisy area [𝝌^2^(1) = 12.12, *p* < 0.001] as barriers to sleep. Males were also more likely to report work related stress as a barrier to treating low mood and depression [𝝌^2^(1) = 3.89, *p* =  0.049]. Female participants were more likely to report that alcohol helped them to cope with stress [χ^2^(1) = 4.51, *p* = 0.034]. Male participants were more likely to report a lack of close friends as being a barrier to engaging in mentally stimulating activity [χ^2^(1) = 5.56, *p* = 0.018].

**Table 3:** Effect of gender on exposure to different barriers.

Risk factor	Exposed (%)	Exposed (n)		Lack of motivation	Emotional	Social	Practical	Lack of knowledge			Barriers (% from exposed to risk factor)							
**Unhealthy diet**	60.4%	333	I have no motivation	Healthy foods do not satisfy my appetite	Stress from work / personal life	Healthy foods take too long to prepare	Healthy foods cost too much	Not like the taste of healthy foods	I am unsure about what a healthy diet is	People I live with
	male	160	61.3%	43.1%	*31.3%	31.3%	33.8%	29.4%	28.8%	**15.0%
	female	173	66.5%	42.2%	*43.4%	32.4%	28.9%	24.3%	24.3%	**28.3%
**Low social interaction**	54.8%	302	Do not socialise much as I like being by myself	Worried about COVID-19	Not many friends	No access to facilities to meet	Live too far away	Cannot afford the costs	Stress meeting others	Not enough time
	male	147	53.1%	46.3%	*55.1%	46.3%	*53.1%	36.1%	35.4%	23.1%
	female	155	56.8%	52.9%	*43.2%	47.7%	*40.0%	42.6%	34.8%	25.8%
**Low physical activity**	39.0%	215	No drive to do it	Gym too expensive	No access to facilities	Physical disability	Home commitments	Work commitments		
	male	109	73.4%	*38.5%	31.2%	33.9%	26.6%	22.9%		
	female	106	82.1%	*51.9%	37.7%	32.1%	34.0%	21.7%		
**Poor quality sleep**	33.4%	184	Too stressed	Do not know why	Family commitments	Caffeine intake	Work commitments	Noisy area		
	male	72	72.2%	58.3%	18.1%	*25.0%	**20.8%	***20.8%		
	female	112	68.8%	68.8%	18.8%	*12.5%	**6.3%	***4.5%		
**Low mood and depression**	27.9%	154	Life at home	Financial worries	Health worries	Worried about asking others	Bad previous experiences with health professionals	Work related stress	Not know how to access services	
	male	64	59.0%	50.8%	52.5%	37.7%	31.1%	*34.4%	23.0%	
	female	90	54.8%	48.8%	46.4%	36.9%	35.7%	*20.2%	23.8%	
**Smoking**	22.7%	125	Not have the willpower to stop	Stress from work or personal life	Other people I live with continue to smoke	Social life I have				
	male	70	64.3%	44.3%	40.0%	28.6%				
	female	55	72.7%	49.1%	45.5%	20.0%				
**Alcohol consumption**	21.6%	119	Not feeling I need to reduce alcohol consumption	Alcohol helps me cope with stress	People around me continue to drink	Social life I have				
	male	85	56.5%	*29.4%	20.0%	17.6%				
	female	34	55.9%	*50.0%	20.6%	14.7%				
**Low mental stimulation**	21.1%	116	Not entertaining	No close friends	Lack of time	Financial means				
	male	68	63.2%	*51.5%	35.3%	32.4%				
	female	48	47.9%	*29.2%	37.5%	37.5%				
**Air pollution**	15.1%	83	Could afford to change the area I live or work in							
	male	46	30.4%							
	female	37	29.7%							

Numbers in bold show significant group differences. ^**^Sig. different at *p* < 0.01, ^***^Sig. different at *p* < 0.001. NB, missing risk factors are due to low n which makes Z-tests not applicable.

### 3.5. Effect of age on exposure to barriers

Table 4 illustrates the effect of age on exposure to barriers. Younger participants were more likely to report stress from their work or personal life as being a barrier to consuming a healthy diet [𝝌^2^(1) = 17.08, *p* < 0.001]. Younger participants were more likely to report multiple barriers to engaging in social activity, namely not having many friends [𝝌^2^(1) = 4.09, *p* = 0.04], being unable to afford the cost [𝝌^2^(1) = 15.92, *p* < 0.001], being stressed by meeting others [𝝌^2^(1) = 4.79, *p* = 0.03], and not having enough time [𝝌^2^(1) = 4.16, *p* = 0.04]. With regard to physical activity, younger participants were more likely to report home commitments as being a barrier [𝝌^2^(1) = 7.71, *p* = 0.006]. Younger participants were more likely to report stress as being a barrier to sleep [𝝌^2^(1) = 8.29, *p* = 0.004], whereas older participants were more likely to be unable to identify a specific barrier [𝝌^2^(1) = 3.88, *p* = 0.049]. Younger participants were more likely to identify work related stress as being a potential cause of low mood and depression [𝝌^2^(1) = 5.91, *p* = 0.015]. Younger participants were more likely to report not having the willpower to stop [𝝌^2^(1) = 13.46, *p* < 0.001], and stress from work or personal life [𝝌^2^(1) = 4.15, *p* = 0.04] as barriers to smoking cessation. No statistically significant differences between age groups were noted for barriers pertaining to alcohol consumption, mentally stimulating activity and air pollution.

**Table 4:** Effect of age on exposure to different barriers.

Risk factor	Exposed (%)	Exposed (n)		Lack of motivation	Emotional	Social	Practical	Lack of knowledge			Barriers (% from exposed to risk factor)							
**Unhealthy diet**	60.4%	333	I have no motivation	Healthy foods do not satisfy my appetite	Stress from work / personal life	Healthy foods take too long to prepare	Healthy foods cost too much	Not like the taste of healthy foods	I am unsure about what a healthy diet is	people I live with
	50-64 y.o.	278	65.1%	42.1%	***42.4%	31.7%	31.3%	29.1%	24.5%	21.9%
	65+ y.o.	55	58.2%	45.5%	***12.7%	32.7%	30.9%	31.5%	36.4%	21.8%
**Low social interaction**	54.8%	302	Do not socialise much as I like being by myself	Worried about COVID-19	Not many friends	No access to facilities to meet	Live too far away	Cannot afford the costs	Stress meeting others	Not enough time
	50-64 y.o.	248	56.8%	48.0%	*52.0%	46.4%	44.4%	***44.8%	*37.9%	*27.0%
	> 65 y.o.	54	47.8%	57.4%	*35.2%	50.0%	55.6%	***14.8%	*22.2%	*13.0%
**Low physical activity**	39.0%	215	No drive to do it	Gym too expensive	No access to facilities	Physical disability	Home commitments	Work commitments		
	50-64 y.o.	170	80.0%	47.6%	34.7%	32.9%	**34.7%	24.7%		
	> 65 y.o.	45	68.9%	35.6%	33.3%	33.3%	**13.3%	13.3%		
**Poor quality sleep**	33.4%	184	Too stressed	Do not know why	Family commitments	Caffeine intake	Work commitments	Noisy area		
	50-64 y.o.	158	***74.1%	*63.3%	19.0%	19.0%	12.7%	12.0%		
	> 65 y.o.	26	***46.2%	*73.1%	15.4%	7.7%	7.7%	3.8%		
**Low mood and depression**	27.9%	154	Life at home	Financial worries	Health worries	Worried about asking others	Bad previous experiences with health professionals	Work related stress	Not know how to access services	
	50-64 y.o.	139	56.2%	52.3%	48.5%	36.9%	35.4%	***29.2%	23.8%	
	> 65 y.o.	15	60.0%	26.7%	53.3%	40.0%	20.0%	***0%	20.0%	
**Smoking**	22.7%	125	Not have the willpower to stop	Stress from work or personal life	Other people I live with continue to smoke	Social life I have				
	50-64 y.o.	112	***73.2%	*49.1%	43.8%	25.0%				
	> 65 y.o.	13	***23.1%	*23.1%	30.8%	23.1%				
**Alcohol consumption**	21.6%	119	Not feeling I need to reduce alcohol consumption	Alcohol helps me cope with stress	People around me continue to drink	Social life I have				
	50-64 y.o.	99	54.5%	38.4%	19.2%	16.2%				
	> 65 y.o.	20	65.0%	20.0%	25.0%	20.0%				
**Low mental stimulation**	21.1%	116	Not entertaining	No close friends	Lack of time	Financial means				
	50-64 y.o.	102	55.9%	40.2%	36.3%	34.3%				
	> 65 y.o.	14	64.3%	57.1%	35.7%	35.7%				
**Air pollution**	15.1%	83	Could afford to change the area I live or work in							
	50-64 y.o.	73	31.5%							
	> 65 y.o.	10	20%							

Numbers in bold show significant group differences. ^*^Sig. different at *p* < 0.05, ^**^Sig. different at *p* < 0.01, ^***^Sig. different at *p* < 0.001. NB, missing risk factors are due to low n which makes Z-tests not applicable.

### 3.6. Effect of household income on exposure to barriers

Table 5 illustrates the effect of household income on exposure to barriers. No statistically significant differences between income groups were noted for barriers pertaining to smoking, alcohol consumption, low mental stimulaton, or air pollution. Participants with lower household income were more likely to report lack of motivation as a barrier to consuming a healthy diet [𝝌^2^(1) = 3.98, *p* = 0.046]. With regard to engaging in social activity, participants with lower household income were more likely to report liking being by oneself [χ^2^(1) = 6.69, *p* = 0.01] whereas those with higher household income were more likely to report not having enough time [χ^2^(1) = 4.24, *p* = 0.04]. Participants with greater household income were more likely to report home commitments [χ^2^(1) = 10.25, *p* = 0.001] and work commitments [𝝌^2^(1) = 13.59, *p* < 0.001] as barriers to undertaking physical activity. Work commitments were also more commonly reported as a barrier to sleep by participants with greater household income [𝝌^2^(1) = 13.36, *p* < 0.001]. With regard to low mood and depression, life at home [𝝌^2^(1) = 9.12, *p* = 0.002], and financial worries [𝝌^2^(1) = 4.32, *p* = 0.04] were more commonly reported as barriers by those with lower household income. Work-related stress was more commonly reported by those with greater household income as a potential cause of low mood and depression [𝝌^2^(1) = 16.53, *p* < 0.001].

**Table 5:** Effect of household income on exposure to different barriers.

Risk factor	Exposed (%)	Exposed (n)		Lack of motivation	Emotional	Social	Practical	Lack of knowledge			Barriers (% from exposed to risk factor)							
**Unhealthy diet**	60.4%	333	I have no motivation	Healthy foods do not satisfy my appetite	Stress from work / personal life	Healthy foods take too long to prepare	Healthy foods cost too much	Not like the taste of healthy foods	I am unsure about what a healthy diet is	people I live with
	≤ €40000/year	179	*68.2%	40.8%	36.9%	30.7%	35.8%	26.8%	30.2%	22.3%
	> €40000/year	118	*56.8%	45.8%	39.8%	33.9%	27.1%	28.0%	22.0%	23.7%
**Low social interaction**	54.8%	302	Do not socialise much as I like being by myself	Worried about COVID-19	Not many friends	No access to facilities to meet	Live too far away	Cannot afford the costs	Stress meeting others	Not enough time
	≤ €40000/year	178	**60.8%	48.3%	46.6%	48.9%	40.4%	42.7%	40.4%	*18.5%
	> €40000/year	99	**49.0%	48.5%	50.5%	44.4%	52.5%	33.3%	29.3%	*30.3%
**Low physical activity**	39.0%	215	No drive to do it	Gym too expensive	No access to facilities	Physical disability	Home commitments	Work commitments		
	≤ €40000/year	122	78.7%	48.4%	36.1%	37.7%	***23.0%	***14.8%		
	> €40000/year	71	76.1%	42.3%	31.0%	25.4%	***45.1%	***38.0%		
**Poor quality sleep**	33.4%	184	Too stressed	Do not know why	Family commitments	Caffeine intake	Work commitments	Noisy area		
	≤ €40000/year	115	73.0%	60.9%	21.7%	20.0%	***6.1%	13.0%		
	> €40000/year	50	68.0%	72.0%	16.0%	18.0%	***28.0%	10.0%		
**Low mood and depression**	27.9%	154	Life at home	Financial worries	Health worries	Worried about asking others	Bad previous experiences with health professionals	Work related stress	Not know how to access services	
	≤ €40000/year	105	**65.1%	*56.4%	52.5%	34.2%	32.9%	***15.4%	22.2%	
	> €40000/year	41	**37.5%	*37.2%	45.6%	44.9%	37.7%	***47.7%	24.6%	
**Smoking**	22.7%	125	Not have the willpower to stop	Stress from work or personal life	Other people I live with continue to smoke	Social life I have				
	≤ €40000/year	80	68.8%	46.3%	41.3%	22.5%				
	> €40000/year	38	71.1%	50.0%	50.0%	34.2%				
**Alcohol consumption**	21.6%	119	Not feeling I need to reduce alcohol consumption	Alcohol helps me cope with stress	People around me continue to drink	Social life I have				
	≤ €40000/year	60	51.7%	40.0%	20.0%	15.0%				
	> €40000/year	51	58.8%	27.5%	21.6%	21.6%				
**Low mental stimulation**	21.1%	116	Not entertaining	No close friends	Lack of time	Financial means				
	≤ €40000/year	66	60.6%	45.5%	30.3%	39.4%				
	> €40000/year	36	63.9%	33.3%	38.9%	22.2%				
**Air pollution**	15.1%	83	Could afford to change the area I live or work in							
	≤ €40000/year	43	20.9%							
	> €40000/year	33	39.4%							

Numbers in bold show significant group differences. ^*^Sig. different at *p* < 0.05, ^**^Sig. different at *p* < 0.01, ^***^Sig. different at *p* < 0.001. NB, missing risk factors are due to low n which makes Z-tests not applicable.

### 3.7. Effect of education on exposure to barriers

Table 6 illustrates the effect of education on exposure to barriers. No statistically significant differences between education groups were noted for barriers pertaining to healthy diet, sleep, smoking, alcohol consumption, low social interaction, air pollution, or engaging in mentally stimulating activity. Participants with higher levels of educational attainment were more likely to report home [𝝌^2^(1) = 11.19, *p* < 0.001] and work [𝝌^2^(1) = 7.31, *p* = 0.007] commitments as barriers to engaging in physical activity whereas physical disability was more likely to impact those educated to secondary school level [𝝌^2^(1) = 6.10, *p* = 0.013]. With regard to low mood and depression, those educated to secondary school level were less likely to know how to access services [χ^2^(1) = 4.75, *p* = 0.03]. Distrust of healthcare professionals or previous bad experiences were, on the other hand, more likely to impact those of higher educational attainment [𝝌^2^(1) = 7.48, *p* = 0.006].

**Table 6:** Effect of education on exposure to different barriers.

Risk factor	Exposed (%)	Exposed (n)		Lack of motivation	Emotional	Social	Practical	Lack of knowledge			Barriers (% from exposed to risk factor)							
**Unhealthy diet**	60.4%	333	I have no motivation	Healthy foods do not satisfy my appetite	Stress from work / personal life	Healthy foods take too long to prepare	Healthy foods cost too much	Not like the taste of healthy foods	I am unsure about what a healthy diet is	People I live with
	Secondary school	180	63.3%	46.1%	36.1%	28.9%	33.3%	28.9%	27.8%	24.4%
	University	153	64.7%	38.6%	39.2%	35.3%	28.8%	24.2%	24.8%	19.0%
**Low social interaction**	54.8%	302	Do not socialise much as I like being by myself	Worried about COVID-19	Not many friends	No access to facilities to meet	Live too far away	Cannot afford the costs	Stress meeting others	Not enough time
	Secondary school	153	52.4%	53.6%	45.8%	44.4%	42.5%	43.8%	37.3%	23.5%
	University	149	57.8%	45.6%	52.3%	49.7%	50.3%	34.9%	32.9%	25.5%
**Low physical activity**	39.0%	215	No drive to do it	Gym too expensive	No access to facilities	Physical disability	Home commitments	Work commitments		
	Secondary school	126	73.8%	46.8%	34.9%	**39.7%	***21.4%	**15.9%		
	University	89	83.1%	42.7%	33.7%	**23.6%	***42.7%	**31.5%		
**Poor quality sleep**	33.4%	184	Too stressed	Do not know why	Family commitments	Caffeine intake	Work commitments	Noisy area		
	Secondary school	103	68.9%	68.0%	16.5%	16.5%	12.6%	10.7%		
	University	81	71.6%	60.5%	21.0%	18.5%	11.1%	11.1%		
**Low mood and depression**	27.9%	154	Life at home	Financial worries	Health worries	Worried about asking others	Bad previous experiences with health professionals	Work related stress	Not know how to access services	
	Secondary school	94	63.0%	50.0%	52.0%	39.0%	**23.0%	19.0%	*28.0%	
	University	60	63.0%	62.0%	39.0%	35.0%	**44.0%	33.0%	*13.0%	
**Smoking**	22.7%	125	Not have the willpower to stop	Stress from work or personal life	Other people I live with continue to smoke	Social life I have				
	Secondary school	85	68.2%	42.4%	36.5%	25.9%				
	University	40	67.5%	55.0%	55.0%	22.5%				
**Alcohol consumption**	21.6%	119	Not feeling I need to reduce alcohol consumption	Alcohol helps me cope with stress	People around me continue to drink	Social life I have				
	Secondary school	71	57.7%	39.4%	18.3%	15.5%				
	University	48	54.2%	29.2%	22.9%	18.8%				
**Low mental stimulation**	21.1%	116	Not entertaining	No close friends	Lack of time	Financial means				
	Secondary school	74	58.1%	47.3%	36.5%	39.2%				
	University	42	54.8%	33.3%	35.7%	26.2%				
**Air pollution**	15.1%	83	Could afford to change the area I live or work in							
	Secondary school	42	26.2%							
	University	41	34.1%							

Numbers in bold show significant group differences. ^*^Sig. different at *p* < 0.05, ^**^Sig. different at *p* < 0.01, ^***^Sig. different at *p* < 0.001. NB, missing risk factors are due to low n which makes Z-tests not applicable.

### 3.8. Cluster analysis

**Figure 2:**
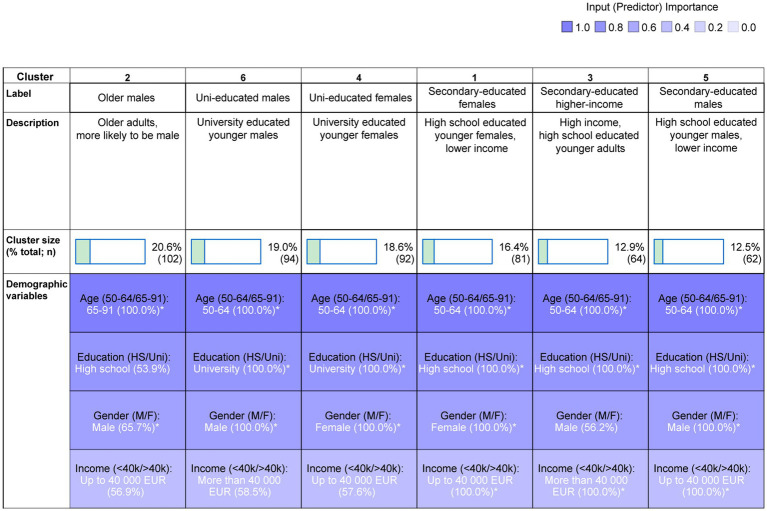
Two-step cluster analysis output and cluster composition. The cluster labels and descriptions are based on the relative contribution of variables within each cluster. *indicates a significant difference from 50/50 distribution.

Cluster analysis revealed six clusters (average Silhouette measure of cohesion and separation = 0.6). Figure 2 shows the cluster size and the demographic variable distribution within each cluster. The colour grading reflects the overall predictor importance (age: 1.00; education = 0.79; gender = 0.68; household income = 0.40). The cluster label is based on the top two most important predictors within each cluster. The cluster descriptions mention all demographic characteristics of the cluster that are significantly more prevalent (i.e., characterise >50% of participants in the cluster). Appendix 2 shows the *z*-tests assessing the significance of this prevalence. Cluster 1 comprised younger females (aged 50–64), who had been educated to secondary school level and had a household income of less than €40,000 (16.4%, *n* = 81); cluster 2 comprised older adults (aged 65–91) who were more likely to be male, educated to high school level, and to have a household income of < €40,000 (20.6%, *n* = 102); cluster 3 comprised secondary school educated, younger adults with high household income, who were more likely to be male (12.9%, *n* = 64); cluster 4 comprised university educated younger females, who were more likely to have low household income (18.6%, *n* = 92); cluster 5 comprised secondary school educated, younger males with low household income (12.5%, *n* = 62); cluster 6 comprised university educated younger males, who were more likely have high household income (19%, *n* = 94).

Table 7 shows the number and proportion of participants within each cluster that have reported being exposed to any of the 13 dementia risk factors. Table 8 shows the significant differences between clusters in the prevalence of each risk factor (with value of *p*s not corrected for multiple comparisons). Several statistically significant differences were detected. Multiple risk factors disproportionately impact those of lower educational status (clusters A, C, and E). Secondary educated males (cluster E) were more likely to report exposure to excess alcohol when compared to other clusters, with the exception of university educated males (cluster F). They were also more likely to report smoking when compared to other clusters, with the exception of secondary educated females (cluster A). Secondary educated females (cluster A) were more likely to report poor sleep when compared to older males (cluster B), Secondary-educated, higher income (cluster C), and Uni-educated males (cluster F).

**Table 7:** Number and proportion of participants within each cluster that have reported being exposed to any of the 13 dementia risk factors.

Risk Factor		Cluster											
		Secondary-educated females		Older males		Secondary-educated, higher income		Uni-educated females		Secondary-educated males		Uni-educated males	
		Count	Column *N* %	Count	Column *N* %	Count	Column *N* %	Count	Column *N* %	Count	Column *N* %	Count	Column *N* %
Diabetes	Present	4	4.9%	11	10.8%	7	10.9%	6	6.5%	4	6.5%	11	11.7%
	Total	81	100.0%	102	100.0%	64	100.0%	92	100.0%	62	100.0%	94	100.0%
Low physical activity	Present	32	42.7%	40	45.5%	26	41.3%	38	46.3%	28	48.3%	29	34.5%
	Total	75	100.0%	88	100.0%	63	100.0%	82	100.0%	58	100.0%	84	100.0%
Sleep problems	Present	40	49.4%	26	25.5%	18	28.1%	33	35.9%	23	37.1%	25	26.6%
	Total	81	100.0%	102	100.0%	64	100.0%	92	100.0%	62	100.0%	94	100.0%
Activities with risk of head injury	Present	0	0.0%	2	2.0%	0	0.0%	3	3.3%	1	1.6%	4	4.3%
	Total	81	100.0%	102	100.0%	64	100.0%	92	100.0%	62	100.0%	94	100.0%
Hearing problems	Present	10	12.3%	19	18.6%	8	12.5%	8	8.7%	9	14.5%	9	9.6%
	Total	81	100.0%	102	100.0%	64	100.0%	92	100.0%	62	100.0%	94	100.0%
High blood pressure	Present	25	30.9%	50	49.0%	25	39.1%	30	32.6%	26	41.9%	31	33.0%
	Total	81	100.0%	102	100.0%	64	100.0%	92	100.0%	62	100.0%	94	100.0%
Overweight	Present	56	71.8%	48	49.5%	45	72.6%	58	63.0%	32	53.3%	58	63.0%
	Total	78	100.0%	97	100.0%	62	100.0%	92	100.0%	60	100.0%	92	100.0%
Smoking	Present	26	32.1%	13	12.7%	17	26.6%	16	17.4%	27	43.5%	19	20.2%
	Total	81	100.0%	102	100.0%	64	100.0%	92	100.0%	62	100.0%	94	100.0%
Social isolation	Present	48	59.3%	50	49.0%	31	48.4%	58	63.0%	36	58.1%	54	57.4%
	Total	81	100.0%	102	100.0%	64	100.0%	92	100.0%	62	100.0%	94	100.0%
Low mental stimulation	Present	18	22.2%	11	10.8%	15	23.4%	18	19.6%	22	35.5%	18	19.1%
	Total	81	100.0%	102	100.0%	64	100.0%	92	100.0%	62	100.0%	94	100.0%
Air pollution	Present	10	12.3%	8	7.8%	13	20.3%	16	17.4%	13	21.0%	16	17.0%
	Total	81	100.0%	102	100.0%	64	100.0%	92	100.0%	62	100.0%	94	100.0%
Alcohol	Present	10	19.6%	19	27.1%	17	28.8%	13	21.3%	27	50.9%	25	38.5%
	Total	51	100.0%	70	100.0%	59	100.0%	61	100.0%	53	100.0%	65	100.0%
Low mood/ depression	Present	36	44.4%	15	14.7%	20	31.3%	31	33.7%	24	38.7%	20	21.3%
	Total	81	100.0%	102	100.0%	64	100.0%	92	100.0%	62	100.0%	94	100.0%

**Table 8:** Each cluster is coded by a letter (1 = A, 2 = B, 3 = C, 4 = D, 5 = E, and 6 = F).

Risk factor		Cluster					
		Secondary-educated females	Older males	Secondary-educated, higher income	Uni-educated females	Secondary-educated males	Uni-educated males
		(A)	(B)	(C)	(D)	(E)	(F)
Air pollution			B(0.019)	B(0.044)	B(0.015)	
Alcohol					A(0.001) B(0.007) C(0.017) D(0.001)	A(0.028) D(0.036)
Low mood/ depression	B(0.000) F(0.001)		B(0.011)	B(0.002)	B(0.000) F(0.018)	
Diabetes						
Low physical activity						
Sleep problems	B(0.001) C(0.009) F(0.002)					
Activities with risk of head injury	.		.			
Hearing problems		D(0.046)				
High blood pressure		A(0.013) D(0.020) F(0.023)				
Overweight	B(0.003) E(0.025)		B(0.004) E(0.028)			
Smoking	B(0.001) D(0.024)		B(0.024)		B(0.000) C(0.046) D(0.000) F(0.002)	
Social isolation				B(0.050)		
Low mental stimulation	B(0.035)		B(0.029)		B(0.000) D(0.027) F(0.022)	

When a risk factor is more prevalent within a cluster, the letter shows which other clusters have lower prevalence and the number in brackets shows the value of *p* of the difference.

## 4. Discussion

In this study, we investigated barriers to lifestyle change to improve brain health in an Irish sample of adults aged 50 and above. Detailed subtyping of barriers, as well as examination of differences according to age, gender, education and income were undertaken, highlighting the diverse nature of barriers to brain health behaviours. Awareness of, and exposure to, modifiable risk factors for dementia, in addition to their associations with gender, age, and education were examined elsewhere and demonstrate a lack of awareness amongst our study population that dementia could be prevented through lifestyle modifications (Dukelow et al., 2023). Increasing knowledge in this regard has been identified as a potential priority in Ireland’s National Dementia public awareness campaign (Hickey, 2019). Effective dementia risk reduction approaches are likely to encompass individual and population focused approaches. In clinical contexts, it is anticipated that evolving memory clinics and dedicated brain health services will fulfil a significant role in dementia risk assessment, communication and delivery of personalised prevention (Frisoni et al., 2023). Our study highlights the individualised nature of risk factor profiles, and barriers to brain health behaviours, thereby underlining the utility of devising personalised risk reduction plans. Beyond secondary prevention, shifting the focus to encompass primary prevention in the general population is vital if dividends are to be seen at a population level (Milne et al., 2021). As well as increasing awareness of modifiable risk factors, we posit the argument that, in order to optimise their impact, public health interventions, rooted in the sociocultural contexts of their intended recipients, should target relevant barriers to risk reduction behaviours. It is recognised that homogeneous public health messages might not be persuasive to heterogeneous audiences (Wakefield et al., 2010). The heterogeneity of barriers to brain health behaviours revealed in this study further highlights that messaging may be irrelevant if it fails to take into account the gender, age, educational status, and income of its target audience. These points are further developed below.

In this study, clusters defined by lower socio-economic status (SES; as indicated by lower education and household income, namely clusters A and E) were disproportionately impacted by multiple risk factors, and several barriers were unequally distributed across socioeconomic groups. SES is a latent construct, comprising measures of income, education and occupation, or some combination of these factors (Baker, 2014). In a global context, income inequality has increased in most high-income countries, and in some middle-income countries since 1990 (United Nations Department of Economic and Social Affairs, 2020). Lower SES is associated with a 2.1-year reduction in life expectancy (Stringhini et al., 2017), and in its own right has been recognised as a barrier to healthy behaviour at mid-life (Kelly et al., 2016). Those of lower SES have a higher risk of dementia, a difference which is explained in part by modifiable risk factors (Deckers et al., 2019). From a dementia perspective, it is known that individuals with higher household income may benefit from earlier diagnosis (Petersen et al., 2021). In our study, those of lower household income were more likely to report lack of motivation as a barrier to consuming a healthy diet. Liking being by oneself was more likely to represent a barrier to social activity in those with lower household income. Home and work commitments, on the other hand, were more likely to represent a barrier to physical activity in those with higher household income. It has been suggested that effective public health programmes must take the needs of those with lower SES into account when designing interventions for dementia prevention at individual and societal level (Deckers et al., 2019). We further postulate that large scale interventions targeted at those of lower SES should be tailored to account for the barriers to risk reduction behaviours which disproportionately impact this cohort. Public health interventions, if they do not account for sociocultural contexts, may paradoxically serve to worsen health inequalities (Stephens et al., 2012). Behaviour change interventions for low-income groups have, however, demonstrated positive effects on physical activity, smoking, and healthy eating (Bull et al., 2014), all of which represent modifiable risk factors for dementia. Specific behavioural change techniques and delivery/context components may significantly increase the effectiveness of interventions targeting healthy eating and physical activity in low-income groups (Bull et al., 2018). The investigation of interventions targeting the broader spectrum of modifiable risk factors for dementia in lower SES cohorts, and clarification of individual components conferring effectiveness, represent priorities in the field of brain health research.

Educational status is significantly associated with awareness of multiple modifiable risk factors for dementia (Dukelow et al., 2023). In the context of dementia, education has long been thought to impact cognitive reserve, reducing susceptibility to age-related changes and Alzheimer’s Disease (AD) pathology (Stern, 2012). Higher educational status appears to impact dementia risk indirectly through its relationship with higher wealth and better lifestyle (Deckers et al., 2019). Lower educational attainment in women, however, is independently associated with an increased risk of dementia-related death (Russ et al., 2013). In our study, individuals of lower educational status were more likely to report a physical disability as a barrier to engaging in physical activity. This cohort were also more likely to report lack of knowledge with regard to accessing services as a barrier to treatment of low mood and depression. Individuals educated to undergraduate level or higher were more likely to report a range of practical barriers. Education is thought to impact health via multiple potential pathways (Cohen and Syme, 2013). Whilst the relationship between education and health behaviours such as smoking, alcohol consumption, and exercise is explained in part by differences in health knowledge, most effects remain after differences in knowledge are controlled for (Kenkel, 1991). Amongst other factors, attending university education is associated with a range of preventative health behaviours (Folta et al., 2009). More broadly speaking, as opposed to relating to quantity, health benefits may relate to the delivery of quality education (Cohen and Syme, 2013). The relationship between educational status, exposure to modifiable risk factors, and broader health outcomes has profound policy implications. Educational inequality has long been a theme in Irish educational discourse (Jeffers and Lillis, 2021). A policy central to tackling educational disadvantage in an Irish context is ‘Delivering Equality of Opportunity in Schools’ (DEIS). Whilst DEIS has demonstrated consistent success in improving academic outcomes across all grade levels (Kavanagh et al., 2017), considerable disparities persist. The consistent prioritisation of educational equity in national policy may ultimately have unintended benefits on health outcomes, potentially impacting dementia risk.

Our study highlighted multiple gender differences across barriers to brain health behaviours. Gender based inequalities related to dementia are widespread (Andrew and Tierney, 2018; Bartlett et al., 2018). Globally, females are disproportionately impacted by dementia. Dementia incidence rates are higher amongst women, with a particular divergence in incidence after the age of 80 (Beam et al., 2018). Disability adjusted life years (DALYs) due to dementia are approximately 60% higher amongst women than men (WHO, 2022). In our study, certain barriers disproportionately impacted female participants. Females were more likely to report particular emotional factors as being barriers to consuming a healthy diet and reducing alcohol intake. The disproportionate impact of stress in these contexts is broadly in keeping with previous work highlighting a tendency for women to relate stress to family and health-related events more frequently than men (Matud, 2004). Females were less likely to report practical factors as barriers to getting a good night’s sleep. At a population health level, designing risk reduction strategies to target gender-specific differences in reported barriers may confer additional impact. Previously, gender has been neglected in many National Dementia Strategies (Bartlett et al., 2018). Despite dementia’s disproportionate impact on those of female gender, it has been suggested that women’s focused dementia research is underfunded. Only 12% of the $2.398 billion 2019 NIH Alzheimer’s disease budget went to women-focused research (Baird et al., 2021). Gender-sensitive public health practise is a necessity, and recent years have seen delineation of implementation strategies at European level (Oertelt-Prigione et al., 2017). Successful examples of gender-sensitive public health interventions can be identified (Gaston et al., 2007; Folta et al., 2009), and may provide a basis for addressing gender-specific differences in brain health behaviours and associated barriers. The StrongWomen—Healthy Hearts programme for example has demonstrated efficacy in reducing cardiovascular disease risk in sedentary midlife and older women who were overweight or obese (Folta et al., 2009), and may provide a template for gender specific dementia risk reduction interventions.

Lack of motivation was the most often cited barrier across multiple cardiovascular risk factors (healthy diet, physical activity, smoking and alcohol excess). Cardiovascular risk factors were prevalent amongst the study population (Table 2). Whilst mortality related to stroke (Soto et al., 2021), and coronary heart disease (Marasigan et al., 2020) in Ireland are falling, the prevalence of some cardiovascular risk factors is increasing (Marasigan et al., 2020) and globally the burden of cardiovascular disease attributable to modifiable risk factors continues to increase (Roth et al., 2020). Burgeoning evidence supports a link between individual cardiovascular risk factors and Alzheimer’s disease dementia (Takeda et al., 2020). Whilst improvements in cardiovascular risk factor control may account for falling dementia prevalence in high-income countries, we demonstrate that cardiovascular risk factors remain prevalent in Ireland. Engaging and motivating those disinclined to participate may be hard to achieve via a public health campaign. In this context, the high prevalence of lack of motivation as a barrier to cardiovascular risk factor control in our study is concerning. Many social psychological models applied in a health behaviour context assume a degree of intrinsic motivation (Hardcastle et al., 2015). Whilst campaigns with mass media components aimed at increasing physical activity have yielded short-term increases, such increases are mainly in highly motivated individuals (Wakefield et al., 2010).

We have stressed the importance of tailored health communication in dementia risk reduction interventions. Tailored health communication may demonstrate increased personal relevance, thereby increasing intent to engage in behavioural change (Bol et al., 2020). Tailored health messaging aside, the authors advocate for the design, implementation, and evaluation of preventive, personalised medicine interventions targeting modifiable risk factors for dementia, and their associated barriers. Ideally, such studies would incorporate measures of genetic, and pathological risk which confer a far higher relative risk than the lifestyle factors discussed herein (Frisoni et al., 2023). The optimal means of evaluating such complex interventions is unclear and may no longer comprise traditional gold standard study designs such as randomised, controlled trials (Garton et al., 2022). Garton et al. (2022) highlight that complex interventions require complex evaluations, underlining the potential role of a non-standard research methodology, namely transdisciplinary research (TDR), in this context. Whilst definitions of TDR vary, common characteristics include, amongst others, transcending disciplinary boundaries through a focus on theoretical unity of knowledge; the inclusion of societal actors as process participants; a focus on specific, complex, societally relevant issues; and working in a transformative manner (Lawrence et al., 2022). TDR can influence scientific productivity and capacity, increasing academic output (Grigorovich et al., 2019). As well as impacting prespecified ‘traditional’ patient outcomes, TDR may have broader social impacts which are difficult to capture through traditional techniques such as assessing policy outcomes (Grigorovich et al., 2019). TDR may represent a promising means to tackle the ‘wicked problem’ (Rittel and Webber, 1973) of increasing dementia incidence.

This study is not without its limitations. The study design introduces the potential for selection bias as participants were members of an existing market research panel. Our study population was highly educated, perhaps limiting the degree to which our results are generalisable in a global context. Our exploration of barriers is systematic, and comparatively exhaustive, based as it is on the Behaviour Change Wheel and under-pinning COM-B model (Michie et al., 2011). It is, however, limited by the use of explicit questions and absence of free text responses which might have revealed novel barriers unanticipated by the research team. We thus cannot be sure that certain barriers for each risk factor were not missed. Patient and public involvement in survey design would have further strengthened this study.

Our study’s principal strength comprises its robust and comprehensive design. Existing literature highlights a multitude of barriers to dementia risk reduction behaviours. Lack of knowledge has been highlighted as the main barrier to behavioural change (Curran et al., 2021). Two studies utilising an identical survey instrument in different populations (Heger et al., 2019; Van Asbroeck et al., 2021) highlight lack of knowledge, and lack of motivation as the two most prevalent barriers. Other factors cited included lack of time, difficulty organising, financial reasons, health problems, and ‘other reasons’. Our work is differentiated by the survey construction, and risk factor-specific nature of the barriers contained therein. The results are likely to be useful in guiding public health messaging and intervention design. A range of barriers were explored across multiple risk reduction behaviours. The heterogeneity of barriers to brain health behaviours revealed in this study highlights the necessity to tailor public health interventions to their target population, taking into account the gender, age, educational status, and income of recipients. Our study serves to add weight to one of the stated goals of the WHO Global action plan on the public health response to dementia 2017–2025, namely the need to organise national and local public health and awareness campaigns that are community and culture specific.

## Data availability statement

The raw data supporting the conclusions of this article will be made available by the authors, without undue reservation.

## Ethics statement

Ethical review and approval was not required for the study on human participants in accordance with the local legislation and institutional requirements. Written informed consent for participation was not required for this study in accordance with the national legislation and the institutional requirements.

## Author contributions

TD and SPK were responsible for the concept and study design and were responsible for final review and editorial decisions. KM was responsible for design of the questionnaire on which the survey was based. TD, SPK, and EGL were responsible for amending and expanding the questionnaire. PV assisted with statistical analysis. Study writeup was undertaken by TD and SPK with input from LJ, EGL, PV, and IK. All authors contributed to the article and approved the submitted version.

## Funding

This study received funding from Five Lives SAS/SharpTx Ltd., who sponsored performance of the survey.

## Conflict of interest

This study received funding from Five Lives SAS/SharpTx Ltd., who sponsored performance of the survey. EGL and KM were previously employed by Five Lives SAS/SharpTx Ltd. LJ and PV are currently employed by Five Lives. IK is a paid medical advisor of Five Lives SAS/SharpTx Ltd.

The remaining authors declare that the research was conducted in the absence of any commercial or financial relationships that could be construed as a potential conflict of interest.

## Publisher’s note

All claims expressed in this article are solely those of the authors and do not necessarily represent those of their affiliated organizations, or those of the publisher, the editors and the reviewers. Any product that may be evaluated in this article, or claim that may be made by its manufacturer, is not guaranteed or endorsed by the publisher.
