# Supplementary material for: Barriers to brain health behaviours: results from the Five Lives Brain Health Ireland Survey
Source: Front Psychol. 2023 Aug 24;14:1101514. doi: 10.3389/fpsyg.2023.1101514 (PMC10483831; doi:10.3389/fpsyg.2023.1101514)
Supplement: Supplementary file 2 [file Data_Sheet_2.DOCX]

**Older males:**

age: z-statistic 10.100; Significance level P < 0.0001; 95% CI of observed proportion 96.45% to 100.00%

education: z-statistic 0.788; Significance level P = 0.4308; 95% CI of observed proportion 43.75% to 63.82%

gender: z-statistic 3.171; Significance level P = 0.0015; 95% CI of observed proportion 55.64% to 74.82%

Income: z-statistic 1.394; Significance level  P = 0.1634; 95% CI of observed proportion 46.72% to 66.67%

**Uni educated males:**

age: z-statistic 9.695; Significance level P < 0.0001; 95% CI of observed proportion 96.15% to 100.00%

education: z-statistic 9.695; Significance level P < 0.0001; 95% CI of observed proportion 96.15% to 100.00%

gender: z-statistic 9.695; Significance level P < 0.0001; 95% CI of observed proportion 96.15% to 100.00%

Income: z-statistic 1.648; Significance level P = 0.0993; 95% CI of observed proportion 47.87% to 68.58%

**Uni-educated females:**

age: z-statistic 9.592; Significance level P < 0.0001; 95% CI of observed proportion 96.07% to 100.00%

education: z-statistic 9.592; Significance level P < 0.0001; 95% CI of observed proportion 96.07% to 100.00%

gender: z-statistic 9.592; Significance level P < 0.0001; 95% CI of observed proportion 96.07% to 100.00%

Income: z-statistic 1.458; Significance level P = 0.1449; 95% CI of observed proportion 46.85% to 67.84%

**Secondary-educated females:**

age: z-statistic 9.000; Significance level P < 0.0001; 95% CI of observed proportion 95.55% to 100.00%

education: z-statistic 9.000; Significance level P < 0.0001; 95% CI of observed proportion 95.55% to 100.00%

gender: z-statistic 9.000; Significance level P < 0.0001; 95% CI of observed proportion 95.55% to 100.00%

Income: z-statistic 9.000; Significance level P < 0.0001; 95% CI of observed proportion 95.55% to 100.00%

**Secondary-educated higher-income:**

age: z-statistic 8.000; Significance level P < 0.0001; 95% CI of observed proportion 94.40% to 100.00%

education: z-statistic 8.000; Significance level P < 0.0001; 95% CI of observed proportion 94.40% to 100.00%

gender: z-statistic 0.992; Significance level  P = 0.3212; 95% CI of observed proportion 43.23% to 68.58%

Income: z-statistic 8.000; Significance level P < 0.0001; 95% CI of observed proportion 94.40% to 100.00%

**Secondary-educated males:**

age: z-statistic 7.874; Significance level P < 0.0001; 95% CI of observed proportion 94.22% to 100.00%

education: z-statistic 7.874; Significance level P < 0.0001; 95% CI of observed proportion 94.22% to 100.00%

gender: z-statistic 7.874; Significance level P < 0.0001; 95% CI of observed proportion 94.22% to 100.00%

Income: z-statistic 7.874; Significance level P < 0.0001; 95% CI of observed proportion 94.22% to 100.00%
